# Do Loneliness and Per Capita Income Combine to Increase the Pace of Biological Aging for Black Adults across Late Middle Age?

**DOI:** 10.3390/ijerph192013421

**Published:** 2022-10-17

**Authors:** Steven R. H. Beach, Eric T. Klopack, Sierra E. Carter, Robert A. Philibert, Ronald L. Simons, Frederick X. Gibbons, Mei Ling Ong, Meg Gerrard, Man-Kit Lei

**Affiliations:** 1 Center for Family Research, The University of Georgia, Athens, GA 30602, USA; 2 Department of Psychology, The University of Georgia, Athens, GA 30602, USA; 3 Leonard Davis School of Gerontology, University of Southern California, Los Angeles, CA 90007, USA; 4 Department of Psychology, Georgia State University, Atlanta, GA 30302, USA; 5 College of Medicine, University of Iowa, Iowa City, IA 52242, USA; 6 Department of Sociology, The University of Georgia, Athens, GA 30602, USA; 7 Department of Psychological Sciences, University of Connecticut, Storrs, CT 06269, USA

**Keywords:** DunedinPACE, loneliness, aging, stress, per capita income

## Abstract

In a sample of 685 late middle-aged Black adults (M age at 2019 = 57.17 years), we examined the effects of loneliness and per capita income on accelerated aging using a newly developed DNA-methylation based index: the DunedinPACE. First, using linear, mixed effects regression in a growth curve framework, we found that change in DunedinPACE was dependent on age, with a linear model best fitting the data (b = 0.004, *p* < 0.001), indicating that average pace of change increased among older participants. A quadratic effect was also tested, but was non-significant. Beyond the effect of age, both change in loneliness (b = 0.009, *p* < 0.05) and change in per capita income (b = −0.016, *p* < 0.001) were significantly associated with change in DunedinPACE across an 11-year period, accounting for significant between person variability observed in the unconditional model. Including non-self-report indices of smoking and alcohol use did not reduce the association of loneliness or per capita income with DunedinPACE. However, change in smoking was strongly associated with change in DunedinPACE such that those reducing their smoking aged less rapidly than those continuing to smoke. In addition, both loneliness and per capita income were associated with DunedinPACE after controlling for variation in cell-types.

## 1. Introduction

The desire for social connection reflects a deeply rooted need [1] that may become more pronounced later in life. The availability of social relationships often decreases with age [2], creating vulnerability to perceived social isolation and the feeling that social needs are not being met. This may result in feelings of “loneliness”. Confirming this expectation, a large body of research indicates that loneliness disproportionately affects older adults [3], resulting in the experience of loneliness being both widespread and consequential for older adults [4]. National population estimates indicate that 20–30% of older adults report loneliness or social isolation [5], and that it is often associated with health consequences. In particular, increased loneliness is associated with greater utilization of health care [6], poorer health [7], increased blood pressure and cardiovascular disease [8], as well as increased mortality [9]. Loneliness may also amplify the impact of other stressors on health and health related outcomes [10], with loneliness potentially exacerbating feelings of stress and anxiety, and further promoting inflammation, blood pressure, and negative affect in reaction to other sources of stress [11,12,13]. Some prior research also suggests the possibility of differences between Black and White older adults in the impact of loneliness [14], and confirms the likely importance of loneliness as a risk factor for poorer self-rated health across groups [15], highlighting the need for additional research on the consequences of loneliness among older Black Americans.

Economic strain is also an important stressor that has been found to be related to health outcomes [16,17]. Health effects related to economic hardship and strain [16], as well as with low-income [17] have been noted for Black Americans. Americans in general view financial insecurity as particularly stressful [18], and there is a strong inverse relation between income and rates of morbidity and mortality in the US [19,20] and also internationally [21]. Because both low per capita income and loneliness are often chronic, lasting for years or an entire lifetime, they have ample opportunity to exert deleterious effects on many domains of everyday life as well as on physical health. In addition, they have ample opportunity to influence each other.

### 1.1. The Need for Examination of Within-Person Change

Prior research on the association of loneliness and low per capita income with health has not typically considered the impact of “within-person” change in loneliness and per capita income on “within-person” change in accelerated aging and health, focusing instead on the association of between person differences in loneliness and low per capita income with concurrent or delayed health outcomes. However, examination of “within-person” change can help clarify and support results from studies focused on between-person effects. That is, between-person associations showing that those higher in loneliness or per capita income are also less healthy concurrently or over time can be influenced by time-invariant “third variables” that may lead to covariation at all points in time. Thus, significant between-person effects may not reflect changes that occur within-individual across time and exert an impact on key health outcomes for individuals. This has important implications for the design of preventive interventions based on this research, creating uncertainty as to whether interventions based on between-person effects alone are likely to produce the hoped-for within-subject effect on outcomes. Conversely, examination of within-person changes associated with outcomes address the way in which changes in one’s own loneliness or per capita income is related to changes in one’s own key health indicators. Examination of within-person effects allows each person to serve as their own control, ruling out time-invariant third-variables as potential confounds. Thus far, researchers have not examined whether treating loneliness and per capita income as time-varying social conditions within-person, rather than simply examining between subject differences results in similar or different conclusions about their association with change in health. In part, this is because the type of prospective longitudinal data needed to address such issues is limited. Accordingly, using latent growth curve and parallel process models, we go beyond prior work to determine if within-person changes in loneliness and per capita income predict trajectories (slopes) of within-person change in DunedinPACE, an innovative, DNA methylation-based marker of the pace of aging. This allows us to test the potential for loneliness and low per capita income to serve as potentially modifiable points of intervention to influence change in DunedinPACE and potentially provide an avenue for addressing disparities in healthy aging among middle-age Black adults.

### 1.2. Epigenetic Measurement Is a Muti-Purpose Tool Enhancing the Study of Health Outcomes

Measurement issues have often made direct assessment of health impacts, health behaviors, and pro-inflammatory processes difficult, and have forced researchers to rely on self-reported health and behavior. Recently, there have been developments in the use of methylation markers that allow more direct assessment in each of these domains, allowing researchers to bypass self-report [22,23]. In addition, epigenetic measurement has the potential to help examine mechanisms of effect. For example, one mechanism potentially linking both financial hardship and loneliness to health outcomes is their potential to contribute to the maintenance of problematic health behaviors [24]. Alternatively, they may exert effects through their impact on immune system functioning such as setting the stage for increased chronic inflammation [25] or initiating other changes in innate or acquired immune responses [26]. Epigenetic assessments can help directly test these possibilities.

#### 1.2.1. Advances in the Measurement of Health Impacts: Epigenetic Clocks

Because the endpoints of chronic illness and mortality do not develop quickly, it has been difficult to rigorously examine the long-term health effects of chronic stressors such as loneliness and per capita income on outcomes of interest. The assessment of so-called “epigenetic clocks” has emerged as a solution to this problem. These indices provide a continuous measure of health, wellness, or risk for morbidity and mortality—indicating the extent to which some individuals are biologically older and more frail (or biologically younger and healthier) than their chronological age would suggest. Those experiencing accelerated epigenetic aging (EA) [27] are expected to have poorer health, suggesting that accelerated EA may be a good way to capture weathering effects of chronic stressors [28], provide a practical, non-self-report assessment of health, and provide a continuous window on an individual’s speed of biological aging, yielding indices that are robust predictors of the diseases of old age as well as time to death [29,30,31,32].

As the development of epigenetic indices of aging proceeded, researchers increasingly focused on second generation indices that predicted biological pathology, chronic illness, and mortality (e.g., [31,33]). These second-generation indices outperformed first-generation indices in predicting various indicators of health outcomes, making them more useful for studies of the development of illness, morbidity, and mortality. In the current investigation we focus on a recently developed, third-generation index, which incorporates several additional improvements and is called the DunedinPACE. As we describe below, this index provides several advantages over prior indices, and the current study offers the opportunity to explore several important issues about its application to an older Black adult sample. Specifically, using a sample of 685 late middle-aged Black adults, we examine the DunedinPACE in a population with elevated risk of chronic illness and early morbidity [34]. In the current investigation we apply the DunedinPACE to a somewhat older sample than the sample on which the scale was developed, allowing us to examine its measurement properties across middle and the transition into older-age among Black adults. Finally, because we have DunedinPACE measures from two timepoints we can directly examine average change in DunedinPACE scores, better estimating the association of age with degree of change in DunedinPACE, and examining the impact of change in per capita income and loneliness on change in DunedinPACE.

#### 1.2.2. The Development of the DunedinPACE

Using an extensive longitudinal data set from the Dunedin longitudinal cohort, Belsky et al. [33] developed a “Pace of Aging”, measure to capture changes across multiple clinical and biological assays, creating a single-timepoint biomarker of rate of change across time and multiple health-related domains. Their initial work resulted in an index called DunedinPoAm [33]; however, this measure was limited in the age range covered, and had several technical problems related to the reliability of probes included in the index. To expand its age range and enhance its test–retest reliability, the authors incorporated new data from the Dunedin cohort to extend the follow-up to include a fourth measurement occasion in the fifth decade of life [35]. The authors also restricted DNA-methylation data to exclude probes identified as having poorer reliability [36]. The resulting index also did not include cg05575921, reducing its overlap with this well-known epigenetic index of smoking. The refined index was named DunedinPACE, for Dunedin (P)ace of (A)ging (C)alculated from the (E)pigenome (see [37]). It is this improved measure of pace of aging that is used in the current investigation because it was specifically designed for use in the context of repeated measures—a focus of the current investigation, as well as for use with middle-aged adults. The current investigation also allowed us to directly examine the issue of invariance of rate of change among older adults. Finally, it allowed us to examine the role of smoking on pace of aging, a health behavior that has previously been found to have a substantial association with health changes later in life, using an epigenetic aging index that is not inherently confounded with smoking indicators. Controlling these influences allowed us to more stringently examine the role of loneliness and per capita income on EA.

#### 1.2.3. Advances in the Measurement of Health Behavior: Smoking

Smoking is an established risk factor for myriad complex disorders of aging. Yet, many prior studies of Epigenetic Aging (EA) have shown only modest effects of smoking and drinking on accelerated aging. One potential reason for the observed lack of association between smoking and EA may be reliance on self-report, which may be unreliable in many samples. In particular, decades of studies have established smoking as the leading non-COVID19 related, preventable cause of premature morbidity and mortality [38,39,40]. Despite this, a recent meta-analysis of studies of Epigenetic Aging (EA) has shown only modest, if any, effects of self-reported smoking and drinking on EA indices of accelerated aging and mortality [41]. Conversely, a recent study by Simons et al. [42] found that a reduction in smoking, using non-self-report indicators, was associated with deceleration of aging among older adults.

When self-reported health behavior data are inaccurate, or substantially underestimates a pattern of problematic use, observed associations with resulting EA indices may be artificially suppressed. There is considerable reason for concern that this may happen in many studies examining smoking. For example, although only 8% of Framingham Health Study (FHS) participants with both self-report and genome-wide DNA methylation profiling self-reported current smoking at their Wave 8 assessment, over 50% of them had probe-based assessment of cg05575921 methylation, a generally accepted biomarker of smoking, in the range suggestive of current and/or past smoking [43]. Combined with prior studies, which show high rates of unreliable self-reports of smoking [17,44,45,46,47,48], we believe that there is considerable reason to be concerned that underreporting could affect conclusions regarding the relationship of smoking to accelerated EA, contributing to apparent inconsistencies in the literature. Accordingly, in the current investigation we use level of methylation at cg05575921 as our index of cumulative exposure to cigarette smoke. Because cg05575921 is not included in the DunedinPACE, it has no inherent confounding with pace of aging.

### 1.3. Intrinsic vs. Extrinsic Indices of Aging to Examine System Effects

Because individual differences in cell type distribution may also account for some differences in observed methylation patterns we examined the effect of predictors on so-called “intrinsic” DunedinPACE, i.e., the index value after controlling for cell type variation. We used a procedure to characterize cell-type variation across individuals described by Horvath [49]. EA indices that do not control cell-type variation are typically referred to as “extrinsic” indices of epigenetic aging. The intrinsic indices control for monocytes, natural killer cells, b cells, CD4 T-helper cells, and CD8 T-helper cells. Monocytes and natural killer cells mediate innate immune responses and express genes that result in inflammatory reactions to infection. B cells, CD4 T-helper cells, and CD8 T-helper cells mediate adaptive immune responses and express genes involved in antibody production and antiviral activity [50]. Accordingly, controlling for variation in these cell-types can highlight the extent to which observed effects of predictors on accelerated aging are mediated by shifts in immune functioning. That is, “extrinsic” EA provides an index that reflects changes in methylation present across all cell-types as well as changes in ratios of white blood cell types. Conversely, “intrinsic” EA measures cellular epigenetic aging after controlling differences in blood cell type counts [51]. If a predictor is more strongly related to one of these measures than to the other, that can be informative. For example, a variable is likely exerting its effect through changes in the immune system if it is strongly related to extrinsic EA but is no longer associated after controlling cell-type variation.

### 1.4. Control Variables

It is not customary to control for age using DunedinPACE, because it is already a measure of change over time and is presumed to be invariant across much of adulthood. However, rate of change in DunedinPACE has not been well characterized for older adults. If it is not invariant across later middle-age, it may be necessary to control age in the analyses to better characterize the impact of other potential independent variables. If age is controlled, for both intrinsic and extrinsic indices, larger positive values indicate accelerated aging and so elevated risk for morbidity and mortality. Smaller negative values indicate decelerated aging. If age is not controlled in DunedinPACE, scores above 1 are typically used to indicate accelerated aging and scores below 1 indicate decelerated aging. In addition to age and gender, it has been noted in prior work that relationship status often has an association with health outcomes. In particular, older individuals who are married or in a long-term cohabitating relationship experience better health and live longer than those who lack such a relationship [52,53]. Therefore, we measured relationship status by assessing whether the respondent reported their marital status as 0 = unmarried, 1 = married/cohabiting. Finally, given their robust relationship to epigenetic aging in prior research with other indices of EA [54] we included non-self-report epigenetic indices of smoking and alcohol use as control variables as well.

## 2. Materials and Methods

### 2.1. Sample

We tested hypotheses using data collected at waves 5 (2008) and 8 (2019) from primary caregivers and their romantic partners in the Family and Community Health Study (FACHS), an ongoing longitudinal study of Black American families initiated in 1997. The original FACHS sample consisted of 889 Black American families, each with a 5th grader, living in Georgia or Iowa. The sampling strategy was designed to generate families representing a range of socioeconomic statuses and neighborhood settings (see [55]). At Wave 1, about half of the families resided in Georgia (*n* = 422) and the other half in Iowa (*n* = 467). Primary caregivers were mostly women and their romantic partners were mostly men. Mean age at wave 5 was 48.7 years (SD = 8.35), and 57.8% of the PCs were married or cohabiting. Eleven years later, at wave 8, mean age for caregivers and their romantic partners was 57.1 years (SD = 6.78) and 55.4% were married or cohabiting. The protocol and all study procedures were approved by the Institutional Review Board at the University of Georgia (Title: FACHS IV; Protocol # Study00000172). Computer-assisted interviews conducted at each wave took an average of 2 h to complete. Within two weeks of the psychosocial interviews at wave 5 and wave 8, a certified phlebotomist visited the home and collected four tubes of blood (30 mL) from each consenting participant. Given the logistics of scheduling home visits by phlebotomists, only members of the sample still residing in Georgia or Iowa at waves 5 and 8 were eligible for the blood draws. Blood was obtained from *n* = 506 of the participants at 5. At wave 8, *n* = 480 individuals, were living in the study area and agreed to provide blood, resulting in a total sample who provided data and a blood sample at either wave 5 or wave 8 of *n* = 693. Unfortunately, 7 of these individuals had missing data and had to be dropped from the analysis. This left 685 individuals (480 women and 205 men) who served as the sample for the present study.

### 2.2. Procedures and Measures

#### 2.2.1. Primary Predictors

##### Loneliness

The measure of loneliness was assessed using two items from the UCLA loneliness scale [56] that were assessed at wave 5 and 8: “How often do you feel that you are no longer close to anyone?” and “How often do you feel left out?” Responses ranged from 1 (Never) to 4 (Always). High scores indicate greater loneliness. The relationship between the two items was significant (*r* = 0.43 at wave 5; *r* = 0.59 at wave 8). Although loneliness is correlated with measures of negative affect, it is nonetheless a distinct psychological experience [57].

##### Per Capita Income

Family per capita income was calculated by dividing the total household income by the number of family members [17].

##### DNA Methylation-Based Measures

Genome-wide DNA methylation assessments were conducted by the University of Minnesota Genome Center (http://genomics.umn.edu/, (Minneapolis, MN, USA)) using the Infinium MethylationEpic Beadchip (Illumina, San Diego, CA, USA) according to the manufacturer’s protocol. The resulting IDAT files were securely transferred, and the data DASEN normalized using the *MethyLumi* [58], *WateRmelon* [59] and *IlluminaHumanMethylationEPICanno.ilm10b2.hg19* [60] R packages as per our previous protocols [61]. Sample and probe level quality control of the data were then conducted as previously described [61]. In brief, samples were removed if more than 1% of their probes had detection *p* values of >0.05. Overall, data from 858,924 of the 866,091 probes in the array were retained.

Beta values for each site were calculated using the standard formula where *U* and *M* are the values of the unmethylated and methylated intensity probes (averaged over bead replicates) and *α* = 100 is a correction term to regularize probes with low total signal intensity [62,63]. CpG values were background-corrected using the “noob” method [64].

$$\beta =\frac{M}{U+M+\alpha }$$

##### DunedinPACE

DunedinPace is designed to provide a “speedometer” of aging that reflects physiological change over the past 12 months, with values greater than one indicating accelerated biological aging. That is, there is an expectation that one year of chronological age will, on average, be associated with a value of 1 on the DunedinPACE. The values for the DunedinPACE indices were calculated using the code supplied by the developers at https://github.com/danbelsky, (1 January 2022). DunedinPace is not currently used in clinical applications. However, it is expected to be useful in the future in assessing outcomes of geroprotective interventions for humans.

##### Cigarette Smoking

cg05575921. Methylation sensitive digital PCR (MSdPCR) assessment of cg05575921 methylation, a generally accepted biomarker of smoking [65,66,67], was determined using the same genome-wide DNA methylation data used to construct the Alcohol Index and the DunedinPACE. Status at cg05575921 is expressed as “% methylation” with levels of <80% being strongly predictive of smoking [67]. Status at the cg05575921 index is used in some clinical applications to identify smoking status.

##### Alcohol Index

Methyl DetectR values for alcohol consumption per week were calculated using the code supplied by the University of Edinburgh website (https://www.ed.ac.uk/centre-genomic-medicine/research-groups/marioni-group/methyldetectr)) [68]. In the training sample, alcohol intake was assessed in units per week and was only considered in those who reported that their intake was representative of a normal week. A natural log(units + 1) transformation was applied to reduce skewness. Accordingly, Methyl DetectR values for alcohol consumption are meant to capture level of usual weekly alcohol consumption. Methyl DetectR values for alcohol consumption are not used in clinical applications. 

##### Cell Type Variation

Cell-type composition was estimated using the “EstimateCellCounts” function in the “minfi” Bioconductor package, which is based on the method developed by Houseman and colleagues [69]. Using this approach, the white blood cell-type proportions (CD4+ T cells, CD8+ T cells, Natural Killer cells, B cells and monocytes) in the whole blood specimens used to prepare the DNA were estimated. These cell-type proportions were then used to examine whether associations between DNAm-based aging measures and predictors free of potentially confounding cell-type variation influences (i.e., to identify associations with an intrinsic index of DunedinPACE).

##### Control Variables

At wave 8, marital/cohabitation status was reported as 0 = unmarried, 1 = married/cohabiting.

### 2.3. Analytic Strategy

To test whether the growth trajectory of DunedinPACE was flat across adulthood through middle-age and the transition to older-age, or whether there was some dependence on chronological age, we first examined an unconditional growth model with individually varying times of observation to examine whether there was a significant change in mean level of DunedinPACE over time and whether the shape of change trajectories was suggestive of linear vs. nonlinear growth [70]. To correct for potential non-independence of observations due to some individuals being in couple relationships, we also included random effects for couple relationship. If the variation associated with couple effects was not significant, to simplify the models, we then dropped this random effect when we ran conditional growth models. We used age as the measure of time in the growth curve models. Because age range varied across the two waves of assessment, individually varying times of observation were used, and age was centered at age 30. All data analyses were performed with Stata version 17 (StataCorp, College Station, TX, USA). To examine substantive effects of loneliness and per capita income, growth models with time-varying covariates were used to test hypotheses regarding time-varying social predictors of change (Loneliness and Per capita income) and control variables (e.g., cigarette smoking and alcohol consumption). We also included random effects of initial levels and the linear growth rates. Missing data can be handled in mixed effects models by using maximum likelihood methods, under the assumption that data are missing at random [71]. This method assumes that missing data are randomly distributed and are unrelated to the dependent variable [72]. This assumption is met in the FACHS sample, as missing data are derived from the random attrition associated with a longitudinal design [73]. 

Accordingly, we began by establishing the shape of growth in DunedinPACE over time and examining its association with participant age at baseline to test whether change in DunedinPACE is the same, on average, regardless of age. We then examined the ability of change in loneliness and change in per capita income to account for variation in change in DunedinPACE controlling effects due to age, gender, education, relationship status, cigarette smoking, and alcohol consumption. We then examined the extent to which associations of loneliness and per capita may be accounted for by their effects on immune function by controlling cell-type variation. Finally, we used a dominance analysis to identify the relative importance of each predictor in explain change in DunedinPACE [74].

## 3. Results

### 3.1. Descriptive Findings

The mean and standard deviation for each of the study variables are shown in Table 1. As can be seen, average levels of DunedinPACE increased over time. In addition, both loneliness and per capita income increased markedly with age. Finally, there was no difference, on average, in level of cigarette and alcohol consumption between baseline and 11-year-follow-up. Given that the rates of DunedinPACE, loneliness, and per capita income increased over time, it was appropriate to use growth models to further examine their associations with each other.

### 3.2. Test of Invariance of Change in PACE Relative to Baseline Age

To establish the shape of change in DunedinPACE across age, we next examined an unconditional growth model with individually varying times of observation to examine whether there was significant change in DunedinPACE as a function of age and, if so, whether the change represented linear or nonlinear growth. As can be seen in Table 2, the best fitting growth curve was a positive linear growth function, with a significant effect for linear growth rate as a function of age (b = 0.004, *p* < 0.01). In addition, there was not a significant effect associated with the random effects for couple relationship, suggesting that partner outcomes were independent and that we could drop this term from the subsequent conditional growth models.

**Figure 1:**
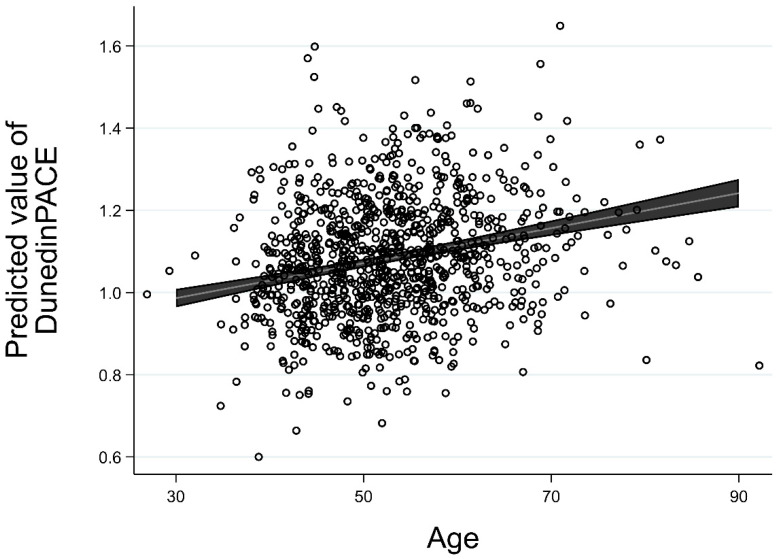
Results from the unconditional linear growth model of DunedinPACE as a function of age, with individual data points shown.

Change in DunedinPACE was expected to have an average value of 1. However, as can be seen in Figure 1, at age 30, average level of DunedinPACE was 0.986, whereas at age 70 it had increased to 1.16. This reflects a significant linear growth rate as a function of baseline age (b = 2.45 × 10^−6^) and underscores that change in DunedinPACE per year of chronological aging was greater for older adults than for younger adults. 

### 3.3. Examination of Loneliness and Per Capita Income

Given the significant effect of age on linear growth in aging, we entered it as a fixed effect in subsequent models. Table 3 presents the parameter estimates predicting linear growth from loneliness, per capita income, control variables and covariates. As can be seen in Model 1A of Table 3, loneliness was included as a time-varying covariate and was positively and significantly associated with linear growth in DunedinPACE, indicating that within-person increases in experiences of loneliness were associated with greater increases in DunedinPACE (b = 0.009, *p* < 0.05). Model 1B shows that this effect was net of the association with change in smoking (b = −0.375, *p* < 0.01), and indicates that continued smoking was also associated with greater acceleration of DunedinPACE.

As can be seen in Model 2A of Table 3, per capita income was included as a time-varying covariate and was negatively and significantly associated with linear growth in DunedinPACE, indicating that greater within-person increases in per capita income were associated with greater decreases in DunedinPACE (b = −0.016, *p* < 0.01). As is shown in Model 2B, this effect was also net of the association with change in smoking (b = −0.358, *p* < 0.01).

Finally, as can be seen in Model 3A of Table 3, when loneliness and per capita income were considered jointly, both had significant effects, suggesting they have independent effects on change in DunedinPACE. In addition, even controlling for cigarette use and alcohol use, both loneliness and per capita income still had significant impact on linear change in DunedinPACE.

We also examined the possibility that significant effects of loneliness and per capita income might be different for men and women by entering interaction terms. However, income x gender and loneliness x gender interaction terms were not significant (b = −0.004, *p* = 0.470 and b = −0.011, *p* = 0.119, respectively), and so were not included in the final model.

### 3.4. Examination of Effects Using Intrinsic DunedinPACE

Finally, we examined the effect of predictors and covariates on “intrinsic” DunedinPACE by controlling cell-type variation. As can be seen in Table 4, change in loneliness and change in per capita remained significant predictors when predicting intrinsic DunedinPACE. Likewise, change in smoking also remained a significant predictor of intrinsic DunedinPACE. Change in cell-type variation was also robustly predictive of linear growth in DunedinPACE, with strong correlations between change in DunedinPACE and change in proportion of CD8+ T cells, CD4+ T cells, Natural killer cells, and B cells, but not monocytes. Using a dominance analysis, we further identified the relative importance of all study variables to DunedinPACE. As can be seen in columns 3 and 4 of Table 4, cg05575921, an indicator of level of cigarette use, is the most prominent and generally dominant predictor of the pace of aging. Loneliness and per capita income contributed approximately 3 to 5 percent of the the within and between individual variation.

## 4. Discussion

DunedinPACE was designed to change in a relatively constant manner across adulthood, with one unit of the PACE reflecting one year of chronological aging on average. However, because DunedinPACE was developed on a sample that was 45 years old at their last assessment, it was unclear whether the scale would show invariance, on average, across the second half of the life span. In addition, given the effects of smoking on health, which may begin to produce a range of systemic problems in the second half of life, it also seemed possible that changes in cigarette smoking would contribute to acceleration or deceleration of DunedinPACE across an 11 year period in later middle-age. Beyond questions about measurement and health behavior correlates, we were also interested in testing hypotheses about the impact of within-person changes in per capita income and loneliness on change in DunedinPACE, i.e., whether DunedinPACE would respond to stresses associated with increased financial difficulties and/or increased loneliness—factors that have affected other DNA methylation-based clocks in between-person comparisons.

In the current set of analyses we found that, for a sample of older middle-age Black Americans, change in DunedinPACE increased significantly with age, with adults over 40 showing significantly more than 1 year of change, on average. Across the age range from age 30 to age 70, PACE of aging showed a significant linear increase such that by age 70 (DunedinPACE = 1.146), participants were aging approximately 16.2% faster, on average, than those who were 30 at baseline (DunedinPACE = 0.986). This may be consistent with other age-related phenotypic changes such as increasing rates of dementia, cardiovascular disease, and mortality, that occur at older ages, and suggests the importance of including chronological age as a covariate in analyses using DunedinPACE, particularly for samples with participants over 40 years old. We also found that, in the current sample, level of continuous cigarette smoking, as indicated by a methylation indicator of smoking (cg05575921), was strongly predictive of change in DunedinPACE across all models.

Even after controlling age at baseline, along with smoking, and other covariates, we found that change in loneliness and per capita income accounted for additional variance in DunedinPACE, reducing the association of age with DunedinPACE. Accordingly, it appears that one mechanism linking greater age at baseline to greater change in DunedinPACE may be its association with an increasing level of loneliness. If so, this may be a targetable point of community-level intervention that could enhance the health of older adults by reducing accelerated aging. Prior work suggests important connections between offspring difficulties during the transition and parent health [75], suggesting that parent-offspring communication may also be associated with loneliness and be another potential target of community-level intervention for older adults.

We also examined the effect of increasing level of per capita income and found that increased per capita income was also associated with decelerated DunedinPACE beyond the effect of all other variables in the model, and it also reduced the association of age with DunedinPACE. Again, this suggests that one mechanism linking greater age at baseline to greater change in DunedinPACE may be its association with increasing per capita income. This may also be a targetable point of community level intervention that could enhance the health of older adults by reducing accelerated aging.

Unlike the effect of chronological age on DunedinPACE, the effect of smoking on DunedinPACE was not diminished by entering loneliness or per capita income as predictors. Likewise, effects were robust to our analysis of intrinsic DunedinPACE, indicating that effects of loneliness and per capita income were not fully explained by their effect on changes in cell-type frequency—a common pathway for extrinsic effects on epigenetic indices of accelerated aging. Accordingly, the current research suggests the hopeful conclusion that programs addressing negative changes in per capita income, preventing increases in loneliness, and encouraging older adults to quit smoking could have substantial potential to enhance healthy aging, potentially protecting the health of those in their fourth decade and beyond.

Limitations. There are also limitations of the current study that are important to note. First, the maximum number of observations for each participant was two, which may have limited our ability to detect curvilinear effects of age on the trajectory of DunedinPACE in this data set. In addition, because our estimates extend only to 70, it is possible that there may be curvilinear effects at older ages. Accordingly, it will be important to replicate the examination of change in older samples with multiple waves of data. Second, the present study utilized a Black American sample. Although this population disproportionately experiences low per capita income and health disparities, future studies should examine whether the same pattern of findings implicating loneliness, low per capita income, and smoking is evident for other racial or ethnic groups as well. Third, the covariates used to capture cell-type variation are indirect estimates based on methylation patterns and so may include substantial error variance. The suggests the potential value of more direct assessment in future research. Finally, the measures of loneliness and per capita income used in this study were obtained from self-reports and, consequently, may be affected by self-report biases. Future work examining these effects using alternative measures of loneliness and per capita income would be helpful in corroborating the current findings.

## 5. Conclusions

Despite its limitations, the current study provides important evidence that change in DunedinPACE escalates with age. This may have important implications for ongoing research and interpretation of a range of findings. Likewise, the current findings indicate that changes in smoking occurring in middle-age can influence change in DunedinPACE, providing support for efforts to attend to health-behavior influences on healthy aging. Finally, the results support the importance of changes in loneliness and per capita income on DunedinPACE net of measurement factors and health behavior, suggesting that these should also be targets of intervention to enhance healthy aging among Black middle-aged adults. 

## Figures and Tables

**Table 1:** Means and standard deviations for study variables.

	2008 (*n* = 495)		2019 (*n* = 478)	
Variables	Mean	SD	Mean	SD
DunedinPACE	1.057	0.143	1.111	0.142
Age	48.765	8.354	57.143	6.781
Loneliness	3.420	1.486	6.730	1.636
Per capita income	13,019.750	13,494.020	19,463.940	17,763.060
Female	0.745	0.436	0.736	0.441
Married/cohabiting	0.578	0.494	0.554	0.498
cg05575921	0.802	0.129	0.801	0.126
Alcohol use	−12.371	0.463	−12.191	0.447
CD8+ T cells	0.090	0.052	0.091	0.052
CD4+ T cells	0.205	0.081	0.180	0.073
Natural killer cells	0.032	0.042	0.031	0.039
B cells	0.085	0.064	0.078	0.055
Monocytes	0.055	0.026	0.064	0.030

**Table 2:** Examination of linear and quadratic models of change in DunedinPACE as a function of age using Unconditional growth models.

	Model 1	Model 2
**Growth factor means**		
Initial status (age 30)	0.986 **	0.963 **
Linear growth rate (per year of age)	0.004 **	0.006 **
Quadratic growth rate		−0.001
**Random variances**		
Initial status (age 30)	0.007	0.007
Linear growth rate (per year of age)	2.45 × 10^−6^	4.25 × 10^−18^
Quadratic growth rate		8.78 × 10^−10^
Couple variance	0.007	0.008
Residual variance	0.005	0.005

† *p* ≤ 0.10; * *p* ≤ 0.05; ** *p* ≤ 0.01 (two-tailed tests).

**Table 3:** Parameter estimates for linear growth models with time-varying and time-invariant covariates examining the separate and joint effects of loneliness and per capita income on DunedinPACE (*n* = 685).

	DunedinPACE					
	Model 1A	Model 1B	Model 2A	Model 2B	Model 3A	Model 3B
Variables	b/(*SE*)	b/(*SE*)	b/(*SE*)	b/(*SE*)	b/(*SE*)	b/(*SE*)
*Fixed effects*						
Initial status	1.014 ** (0.019)	1.136 ** (0.115)	0.981 ** (0.017)	1.146 ** (0.114)	1.006 ** (0.019)	1.099 ** (0.115)
Linear growth rate	0.003 ** (0.001)	0.003 ** (0.001)	0.005 ** (0.001)	0.004 ** (0.001)	0.004 ** (0.001)	0.004 ** (0.001)
*Time-varying covariates*						
Loneliness	0.009 * (0.004)	0.010 ** (0.004)			0.012 ** (0.004)	0.012 ** (0.004)
Per capita income			−0.016 ** (0.004)	−0.011 * (0.004)	−0.017 ** (0.004)	−0.013 ** (0.004)
Married/cohabiting	−0.011 (0.009)	−0.010 (0.009)	−0.006 (0.009)	−0.005 (0.009)	−0.008 (0.009)	−0.006 (0.009)
cg05575921		−0.375 ** (0.039)		−0.358 ** (0.040)		−0.359 ** (0.039)
Alcohol use		−0.013 (0.010)		−0.008 (0.009)		−0.014 (0.010)
*Time-invariant covariates*						
Female	0.001 (0.013)	0.030 * (0.012)	−0.001 (0.013)	0.029 * (0.012)	−0.001 (0.012)	0.028 * (0.012)
*Random effects*						
$\tau_{\left(intercept\right)}$	0.016 *	0.015 *	0.018 *	0.017 *	0.017 *	0.016 *
$\tau_{\left(Age\right)}$	4.31 × 10^−6^	3.25 × 10^−6^	9.26 × 10^−6^	7.00 × 10^−6^	6.72 × 10^−6^	4.75 × 10^−6^
$\tau_{\left(interacept,\;Age\right)}$	−0.001	−0.001	−0.001	−0.001	−0.001	−0.001
$\sigma^{2}$	0.005 *	0.005 *	0.005 *	0.005 *	0.005 *	0.005 *

Note: Unstandardized (b) coefficients shown, with robust standard errors in parentheses. Loneliness and per capita income are standardized by z-transformation (mean = 0 and *SD* = 1). Alcohol use = DNA methylation-based alcohol use. † *p* < 0.10; * *p* < 0.05; ** *p* < 0.01 (two-tailed tests).

**Table 4:** Dominance analysis and parameter estimates for linear growth models with time-varying and time-invariant covariates and cell-types (*n* = 685).

	DunedinPACE			
	Model		Standardized Dominance Weight for within Individual	Standardized Dominance Weight for between Individual
Variables	b	*SE*		
*Fixed effects*				
Initial status	1.273 **	0.106		
Linear growth rate	0.003 **	0.001	0.059	0.035
*Time-varying covariates*				
Loneliness	0.008 **	0.003	0.040	0.030
Per capita income	−0.013 **	0.004	0.044	0.051
Married/cohabiting	−0.003	0.008	0.009	0.010
cg05575921	−0.326 **	0.036	0.286	0.317
Alcohol use	−0.013	0.009	0.003	0.002
CD8+ T cells	−0.520 **	0.078	0.185	0.193
CD4+ T cells	−0.481 **	0.053	0.272	0.268
Natural killer cells	−0.302 **	0.096	0.027	0.027
B cells	−0.170 *	0.066	0.024	0.020
Monocytes	0.265 †	0.140	0.039	0.032
*Time-invariant covariates*				
Female	0.028 *	0.011	0.004	0.015
*Random effects*				
$\tau_{\left(intercept\right)}$	0.010 *			
$\tau_{\left(Age\right)}$	5.07 × 10^−7^			
$\tau_{\left(interacept,\;Age\right)}$	0.001			
$\sigma^{2}$	0.004 *			

Note: Unstandardized (b) coefficients shown, with robust standard errors in parentheses. Loneliness and per capita income are standardized by z-transformation (mean = 0 and *SD* = 1). Alcohol use = DNA methylation-based alcohol use. † *p* < 0.10; * *p* < 0.05; ** *p* < 0.01 (two-tailed tests).

## Data Availability

The data have not been made public; however, the data presented in this study are available to request from the corresponding author.
